# Dynamic CBCT Imaging using Prior Model-Free Spatiotemporal Implicit Neural Representation (PMF-STINR)

**Published:** 2023-11-16

**Authors:** Hua-Chieh Shao, Mengke Tielige, Tinsu Pan, You Zhang

**Affiliations:** Medical Artificial Intelligence and Automation (MAIA) Laboratory, Department of Radiation Oncology, UT Southwestern Medical Center, Dallas, TX, 75235, USA; Medical Artificial Intelligence and Automation (MAIA) Laboratory, Department of Radiation Oncology, UT Southwestern Medical Center, Dallas, TX, 75235, USA; Department of Imaging Physics, University of Texas MD Anderson Cancer Center, Houston, TX, 77030, USA; Medical Artificial Intelligence and Automation (MAIA) Laboratory, Department of Radiation Oncology, UT Southwestern Medical Center, Dallas, TX, 75235, USA

**Keywords:** Dynamic CBCT, image reconstruction, implicit neural representation, data-driven motion modeling

## Abstract

Dynamic cone-beam computed tomography (CBCT) is desired in clinics to capture high-spatial-resolution, time-varying dynamic images, which enable accurate guidance for motion monitoring, patient setup, and adaptive planning of radiotherapy. However, dynamic CBCT reconstruction is a highly ill-posed spatiotemporal inverse problem, as each CBCT volume in the dynamic sequence is only captured by one or a few X-ray projections, due to the slow imaging acquisition speed and the fast anatomical motion (e.g., breathing). To address this challenge, we developed a machine learning-based technique (PMF-STINR) to reconstruct dynamic CBCTs. PMF-STINR employs a joint image reconstruction and registration approach to address the under-sampling challenge, enabling dynamic CBCT reconstruction from conventional 3D CBCT scans. Specifically, PMF-STINR uses spatial implicit neural representation (INR) to reconstruct a reference CBCT volume; and it applies temporal INR to represent the intra-scan dynamic motion with respect to the reference CBCT to yield dynamic CBCTs. To represent time-varying deformable motion, PMF-STINR couples the temporal INR with a B-spline-based, data-driven motion model. The spatial INR, the temporal INR, and the B-spline model of PMF-STINR are all learned on the fly during reconstruction, without using any patient-specific prior knowledge or motion sorting/binning. PMF-STINR was evaluated via digital phantom simulations and physical phantom measurements. It was also evaluated on a multi-institutional dataset of real patient cone-beam projections featuring various imaging protocols. The results showed that the ‘one-shot’ learning-based PMF-STINR makes an accurate and robust reconstruction technique of dynamic CBCTs. It can capture regular/irregular motion with high temporal (~0.1s) resolution and sub-millimeter accuracy.

## I. Introduction

Cone-beam computed tomography (CBCT) is widely used in clinical practice. In radiotherapy, CBCT provides high-spatial-resolution volumetric imaging guidance for treatment setup, dose verification, and adaptive therapy [1]. For CBCT imaging, cone-beam projections are acquired by a source-detector pair that rotates around the patient. The acquisition efficiency is limited by the rotation speed which is generally restricted to ~6°/second (s) for patient safety. Accordingly, it takes ~1 minute (min) to acquire a 360° scan. Due to the long acquisition time, patient anatomical motion, mostly respiration (3–5s per cycle), results in artifacts and blurriness in the reconstructed CBCTs. To address the artifacts and resolve the underlying motion, four-dimensional (4D)-CBCT was developed [2]. 4D-CBCT sorts the projections into a predefined set of motion bins and stacks semi-static CBCTs reconstructed from each bin to represent an averaged motion pattern. The motion sorting assumes that the underlying anatomical motion is periodic and regular, which is in general false [5]. Correspondingly, 4D-CBCT cannot capture irregular motion, which can significantly impact patient setup and dose delivery accuracy [6, 7]. Moreover, 4D motion sorting is usually based on surrogates (e.g., body surface), and can be inaccurate due to limited surrogate-anatomy motion correlation.

A fundamental solution to the limitations of 4D-CBCT is to reconstruct a dynamic sequence of CBCTs (one CBCT for one projection), which eliminates the uncertainties from motion sorting to capture both regular and irregular motion. However, the information captured by each 2D projection is extremely limited for CBCT reconstruction, as conventional reconstruction methods require hundreds of projections. To promote under-sampled reconstruction, previous studies attempted 4D or dynamic CBCT reconstruction by infusing strong assumptions including prior knowledge of the patient anatomy (anatomical model) and/or the motion (motion model). With prior knowledge of the patient anatomy (e.g., a prior CT), techniques were developed to use under-sampled projections to deform the prior CT to 4D-CBCTs, assuming the underlying anatomical model does not change. To enable single projection-based dynamic CBCT reconstruction, a principal component analysis (PCA)-based motion model derived from patient-specific prior 4D-CTs was introduced, to achieve further dimension reduction to satisfy the extreme under-sampling scenario [8, 9]. However, the assumption of the same anatomical model may be invalidated by non-deformation-related anatomical changes over time, for instance, normal tissue inflammation and disease progression. The use of an anatomical model derived from a different machine rather than the same CBCT device also poses additional challenges caused by differing energy/scatter/noise conditions and image intensity variations. The assumption of the same motion model, on the other hand, may not account for inter-fractional motion, or intra-fractional motion pattern variations. The venue to generate such a motion model (e.g., prior 4D-CTs), may contain motion sorting artifacts or not be available for some patients.

In recent years, deep learning (DL)-based approaches were also investigated for severely under-sampled CT/CBCT reconstruction. Shen et al. [10] developed a patient-specific encoder-decoder network to directly map an X-ray projection to a 3D volume. Xiao et al. [11] proposed a cascade framework that combined a dual-attention mechanism and a 4D-CT motion model to reconstruct a CBCT from a single projection. However, the projection-to-CBCT inverse mapping is extremely ill-conditioned, as such models heavily rely on the comprehensiveness of the training dataset. A small out-of-distribution variation, for instance the anatomy/motion change or the imaging parameter change, may result in substantial instabilities from these models. Recently, a machine learning technique named implicit neural representation (INR) has emerged for imaging applications. It uses the non-parametric representation capability of neural networks to learn implicit mapping of complex 3D scenes (e.g., CBCTs) from sparse 2D views (e.g., cone-beam projections) [12]. Acting as a universal function approximator, INR takes geometric coordinates of a scene as inputs and maps them to queried physical features at the coordinates (e.g., linear attenuation coefficients of CBCTs). Compared with conventional voxel-grid-based representation, INR can take non-integer coordinates as inputs for continuous mapping, allowing resolution-agnostic representation of the underlying scene. The continuous, differentiable, and implicit nature of INR allows it to map highly complex medical images in a compact format and promotes image reconstruction from under-sampled signals. Inspired by its potential, we recently developed an INR-based framework, spatial and temporal implicit neural representation (STINR), for dynamic CBCT reconstruction [13]. Compared with previous DL-based approaches that require a large training dataset, STINR reconstructs dynamic CBCTs through directly learning from scan-specific cone-beam projections (‘one-shot’), avoiding potential issues of overfitting and domain shift. By STINR, we decoupled the spatiotemporal dynamic CBCT inverse problem into reconstructing a reference CBCT volume (spatial INR) and the intra-scan motion (temporal INR) related to the reference CBCT, with the help from a patient-specific motion model derived from a prior 4D-CT. However, as mentioned, a high-quality prior 4D-CT may not always be available for motion modeling; and the motion model learnt from it can be outdated and error-prone for following dynamic CBCT reconstruction.

Built on the foundation of STINR [13], in this study we developed a prior model-free STINR (PMF-STINR) framework to solve the above-mentioned challenges in dynamic CBCT imaging. Compared with the original STINR and the other methods, PMF-STINR does not use any prior anatomical or motion model, thus is not prone to the limitations of strong *a priori* assumptions. Instead, it uses a data-driven motion model directly learnt on the fly from the acquired cone-beam projections. Moreover, PMF-STINR does not require any motion sorting/binning of the cone-beam projections, in contrast to the original STINR framework which still needed motion binning in the early stages of reconstruction. Main contributions of this study include:

- The development of a prior model-free approach to jointly reconstruct dynamic CBCTs and solve intra-scan volumetric motion, which eliminates the necessity of motion sorting and reconstructs a CBCT and solves the motion per X-ray projection from conventional 3D CBCT scans.
- The development of a ‘one-shot’ INR-based framework by a multi-staged reconstruction strategy with additional spatiotemporal regulations, allowing dynamic CBCT reconstruction and spatiotemporal mapping without relying on any pre-trained model.
- The comprehensive evaluation and validation of PMF-STINR, including an XCAT [14] simulation study, a dynamic thorax phantom measurement study, and a patient study using multi-institutional clinical cone-beam projections featuring combined anatomical, motion, and imaging variations.

## II. Materials And Methods

### A. Problem formulation

Let ${\left\{p_{t}\right\}}_{t=0}^{N_{p}-1}$ be a consecutive sequence of cone-beam projections of a dynamic subject, where t denotes the frame index labeling the acquisition order of each projection, and $N_{p}$ is the total number of frames. Dynamic CBCT imaging is to reconstruct a sequence of 3D volumes {I(x, t)} from $\left\{p_{t}\right\}$ to represent the dynamic subject. Physically, {I(x, t)} represents the linear attenuation coefficients at spatial coordinates $x\in R^{3}$ and a temporal coordinate t∈R. The reconstruction problem is typically solved within an optimization framework:

(1) $$\left\{\hat{I}\left(x,t\right)\right\}=\underset{\left\{I\left(x,t\right)\right\}}{\text{argmin}}\left(\|𝒫\left\{I\left(x,t\right)\right\}-{\left\{p_{t}\right\}\|}^{2}+\lambda R\right),$$

where 𝒫 denotes the projection matrix, and λ is the weighting factor for an regularization term R. Eq. 1 enforces the projection-domain data fidelity (first term), while R regularizes the image/motion in a transformed domain to avoid overfitting and sub-optimal solutions (e.g., total variation).

Solving the optimization problem of Eq. 1 can be extremely challenging. A whole dynamic sequence {I(x, t)} contains $𝒪\left(10^{8}\right)$ or more voxels to solve, while the volumetric information at each moment t is only captured by a single 2D projection $p_{t}$. However, assuming the underlying anatomy remains unchanged during the scan (which is generally valid under physiological motion), we could use a joint reconstruction and deformable registration approach to simplify the inverse problem. The joint approach de-couples the spatiotemporal inverse problem into reconstructing a reference-frame CBCT $I_{ref}(x)$ and solving an intra-scan motion model with respect to $I_{ref}(x)$, which can be described as a time-dependent deformation vector field (DVF) sequence {d(x, t)}. The whole dynamic CBCT sequence {I(x, t)} can be obtained by deforming/warping the reference CBCT with {d(x, t)}:

(2) $$\left\{I\left(x,t\right)\right\}=I_{\text{ref }}\left(x+\left\{d\left(x,t\right)\right\}\right).$$

To reduce the dimensionality of the solution space for {d(x, t)}, the inherent redundancy of anatomic motion could be further leveraged to yield a low-rank representation of {d(x, t)}. Each time-dependent motion field d(x, t) is approximately separable [15] as a summation of products of spatial $\left(e_{i}(x)\right)$ and temporal $\left(w_{i}(t)\right)$ components:

(3) $$d\left(x,t\right)=\sum_{i=1}^{L}w_{i}\left(t\right)\times e_{i}\left(x\right).$$

These spatial components ${\left\{e_{i}(x)\right\}}_{i=1}^{L}$ maximally capture the motion variations, and compose a basis set (principal motion components, PMC) to explain the motion space. Previous studies, including STINR, assume the PMCs ${\left\{e_{i}(x)\right\}}_{i=1}^{L}$ could be extracted from prior 4D images like 4D-CTs. In contrast, PMF-STINR directly learned such components from the acquired projection data. Considering Eqs. 1–3, the dynamic CBCT reconstruction is equivalent to reconstructing a reference CBCT $I_{ref}$, while finding an optimal time-dependent linear combination of PMCs $\left\{e_{i}(x)\right\}$ that maximally accounts for the underlying anatomical motion. By PMF-STINR, a B-spline-based parametrization of $\left\{e_{i}(x)\right\}$ was employed to further reduce the number of unknowns to $𝒪\left(10^{4}\right)$ (Secs. II.B). As previous studies, PMF-STINR used 3 PMCs for each Cartesian direction (L=3) [8].

### B. PMF-STINR Workflow

**Fig. 1:**
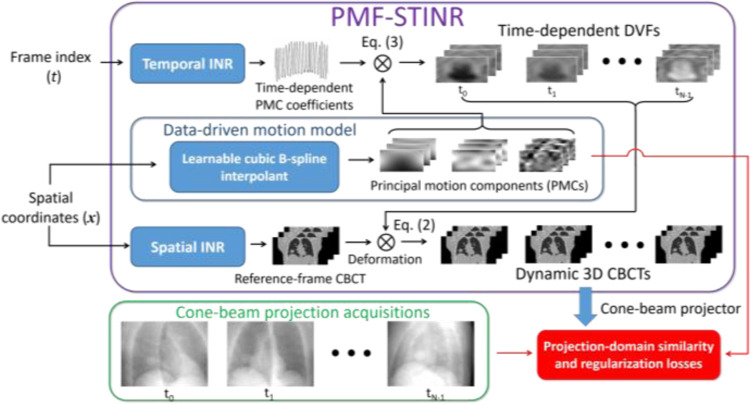
Workflow of PMF-STINR. PMF-STINR solves a sequence of dynamic CBCTs, by using a spatial INR to reconstruct a reference-frame CBCT and a temporal INR as well as a data-driven motion model to represent the intra-scan motion.

Figure 1 illustrates the workflow of PMF-STINR, which comprises three major blocks: spatial INR, temporal INR, and a learnable cubic B-spline interpolant for data-driven motion modeling. The spatial INR reconstructs a reference-frame CBCT of the dynamic sequence, and the temporal INR, in conjunction with the data-driven motion model, solves the time-dependent DVFs of the subject with respect to the reference CBCT. The motion model learns data-specific PMCs from the cone-beam projections, while the temporal INR captures the time-varying temporal coefficients $w_{i}(t)$ for the PMCs. PMF-STINR uses the B-spline to parametrize the dense PMCs with a coarser grid of control points to leverage the piecewise smooth nature of motion fields. The value of control point, representing the motion, is learnable via the PMF-STINR framework.

Via PMF-STINR, the spatial and temporal INRs and the data-driven motion model are jointly trained, reconstructing the reference-frame CBCT and solving the intra-scan motion simultaneously. The network training is driven by both projection-domain data fidelity loss and spatiotemporal regularization losses. In the following subsections we described each component of PMF-STINR and the training strategy.

#### 1) Spatial implicit neural representation

**Fig. 2:**
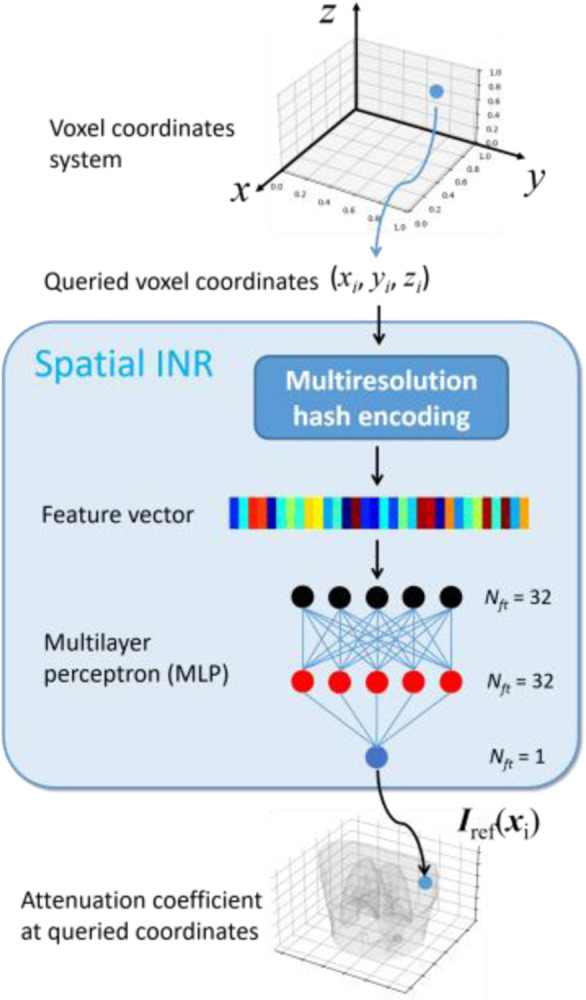
Details of the spatial INR. The spatial INR reconstructs a reference-frame CBCT by learning a continuous mapping from the voxel coordinate system to the linear attenuation coefficient, utilizing multiresolution hash encoding and a multilayer perceptron (MLP).

Figure 2 presents the details of the spatial INR. It takes a voxel coordinate $x_{i}$ of the reference CBCT as input, and maps $x_{i}$ to the attenuation coefficient $I_{\text{ref}}\left(x_{i}\right)$. Before inputting into the spatial INR, the voxel coordinate $x_{i}$ is rescaled to [−1, 1]. The whole reference CBCT can be queried by traversing all voxel coordinates using the same INR. The spatial INR comprises a learnable multiresolution hash encoding step and a multilayer perceptron (MLP) that acts as a non-parametric universal function approximator. As MLP alone is ineffective in learning high-frequency details [16], the hash encoding added prior to the MLP helps to facilitate the fine structure learning [17]. The hash encoding maps a queried coordinate $x_{i}$ to a high-dimensional feature vector, via a multilevel encoding scheme. At each level, a uniform grid of points is set up (denser grids for higher levels), and a predefined hash function [17] maps the neighboring grid points of the queried coordinate $x_{i}$ to the indices of a hash table to retrieve their corresponding feature vectors. Based on the feature vectors of neighboring grids, the feature vector of $x_{i}$ is derived by trilinear interpolation. Finally, the feature vectors of $x_{i}$ at all resolution levels are concatenated and fed into the following MLP. The hash table entries are learnable parameters to handle hash collisions and learn most important feature values [17]. The hash encoding significantly improves the learning efficiency and reduces the needed complexity of the subsequent MLP to map subjects. The recommended hyper-parameters of hash encoding [17] were adopted in this study.

With hash encoding, the MLP of the spatial INR only used three fully connected layers for this study. The feature numbers of the input, hidden, and output layers were 32, 32, and 1, respectively. The periodic activation function, SIREN [18], was used to better capture fine details of CBCT.

#### 2) Temporal implicit neural representation

**Fig. 3:**
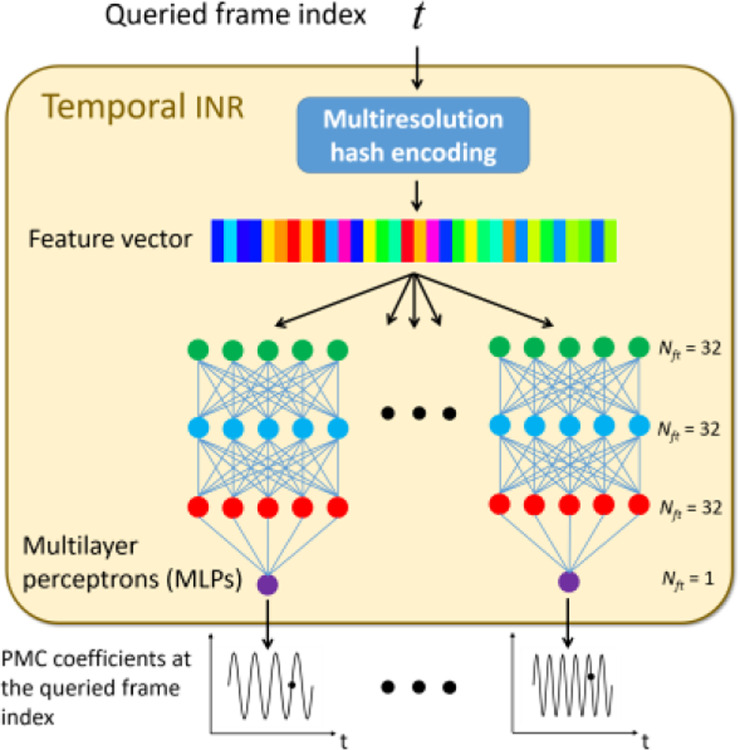
Details of the temporal INR. The temporal INR maps a frame index t to the temporal coefficient $w_{i}(t)$ of the PMCs, using learnable multiresolution hash encoding and nine MLPs. Each of the MLPs corresponds to a Cartesian component of ${\left\{e_{i}(x)\right\}}_{i=1}^{3}$.

The temporal INR captures the temporal variations of the PMC coefficients to represent intra-scan motion (Fig. 3). The input is a frame index t, and the temporal INR outputs the time-dependent coefficients $w_{i}\left(t\right)$ (i=1-3) of the PMCs. Similar to the spatial INR, the frame index t is rescaled between −1 and 1 before feeding into the temporal INR. The whole temporal sequence can be obtained by traversing all frame indices of the acquisition. Similar to the spatial INR, the temporal INR consists of a multiresolution hash encoding step and nine MLPs. Each of the nine MLPs corresponds to a Cartesian component (x, y, z) of the three PMCs, and all MLPs use the same network architecture. In this study, the MLPs comprised one input and one output layer, and two hidden layers. The feature numbers were 32 for the input and hidden layers, and 1 for the output layer. The temporal INR used the same hyper-parameters of the hash encoding as the spatial INR.

#### 3) Data-driven motion modeling

Coupled with the temporal INR (Sec. II.B.2), the data-driven motion model learns the PMCs $\left\{e_{i}(x)\right\}$ of the dynamic DVFs from the cone-beam projections without patient-specific prior knowledge. $\left\{e_{i}(x)\right\}$ are represented by cubic B-spline interpolants, which parametrize the 3D Cartesian space via B-spline interpolations between a coarse, uniform 3D grid of control points. The interpolant takes a form of cubic polynomials, and the first derivative of the interpolant is smooth across the joints of the cubic splines. The global continuity and smoothness are considered desired properties of non-rigid deformations, preventing self-folding of soft tissues and preserving topology. The grid parametrization is computationally efficient and allows dimension reduction, while achieving flexibility with local control of the DVFs. The B-spline-based interpolants are also numerically stable.

Specifically, let ${\left\{\left(s_{i,l}^{x},s_{i,l}^{y},s_{i,l}^{z}\right)\right\}}_{l=0}^{N_{i}}$ denote the coordinates of 3D grid points for $e_{i}(x)$, where $N_{i}$ is the number of grid points along a Cartesian direction (same for all three directions). Let $e_{i,k}$ be the $k^{\text{th}}$ directional component of $e_{i}(x)$, then the 3D B-spline interpolation of $e_{i,k}(x,\;y,\;z)$ at a queried point (x, y, z) is performed via sequential 1D interpolations across the x, y, and z directions. For instance, when the B-spline interpolation was performed in the x-direction, $e_{i,k}$ was written as a linear superposition of cubic B-spline basis functions:

(4) $$e_{i,k}\left(x,s_{i,l^{\prime }}^{y},s_{i,l^{\prime \prime }}^{z}\right)=\sum_{l}P_{i,k}\left(l,l^{\prime },l^{\prime \prime }\right)\times B_{i,l,3}\left(x\right),$$

where l denotes all grid points along the x direction, and $l^{\prime }$ and $l^{\prime \prime }$ are the neighboring grid points of queried y and $z.\;P_{i,k}\left(l,l^{\prime },\;l^{\prime \prime }\right)$ denotes the value of the control point at grid point $\left(s_{i,l}^{x},s_{i,l^{\prime }}^{y},s_{i,l^{\prime \prime }}^{Z}\right)$, and $B_{i,l,3}(x)$ is the cubic basis function which can be derived by the Cox-de Boor recursion formula:

(5) $$B_{i,l,0}\left(x\right)=\{\begin{array}{cc} 1 & \text{ if }s_{i,l}^{x}\leq x<s_{i,l+1}^{x}\\ 0 & \text{ otherwise } \end{array},$$

(6) $$B_{i,l,p}\left(x\right)=\frac{x-s_{i,l}^{x}}{s_{i,l+p}^{x}-s_{i,l}^{x}}B_{i,l,p-1}\left(x\right)+\frac{s_{i,l+p+1}-x}{s_{i,l+p+1}^{x}-s_{i,l+1}^{x}}B_{i,l+1,p-1}\left(x\right).$$

Here p denotes the B-spline degree. After the x-direction interpolation, the y- and z-direction interpolations were performed sequentially in a similar manner. By PMF-STINR, the values of the control points $\left\{P_{l}\right\}$ in Eq. 4 are learnable parameters that characterize the underlying motion. In this study, we adopted the B-spline interpolant model from [19]. The input into the B-spline interpolant model was the voxel coordinates (normalized to [0, 1]), and the model output the vector fields $\left\{e_{i}(x)\right\}$ at the queried coordinates.

The anatomical motion, especially respiration, usually involves deformations across multiple scales. For example, the tissue deformation caused by the diaphragm contraction is typically bulky and large-scale, while the lung nodule motion is more local. To better account for the complexity of motion, PMF-STINR applies a hierarchical multiresolution strategy to represent the motion fields, with similar approaches demonstrated effective in deformable registration to avoid sub-optimal solutions [20]. Via the multiresolution strategy, the three PMCs $e_{i}(x)$ of each Cartesian direction correspond to three different spatial resolutions and motion scales. Each of the three PMCs is represented by a B-spline interpolant of a different grid resolution. The motion fields represented by the PMCs were summed in the end via the related temporal INR coefficients $w_{i}(t)$ to capture both global and local motion.

#### 4) The progressive training strategy

**Fig. 4:**
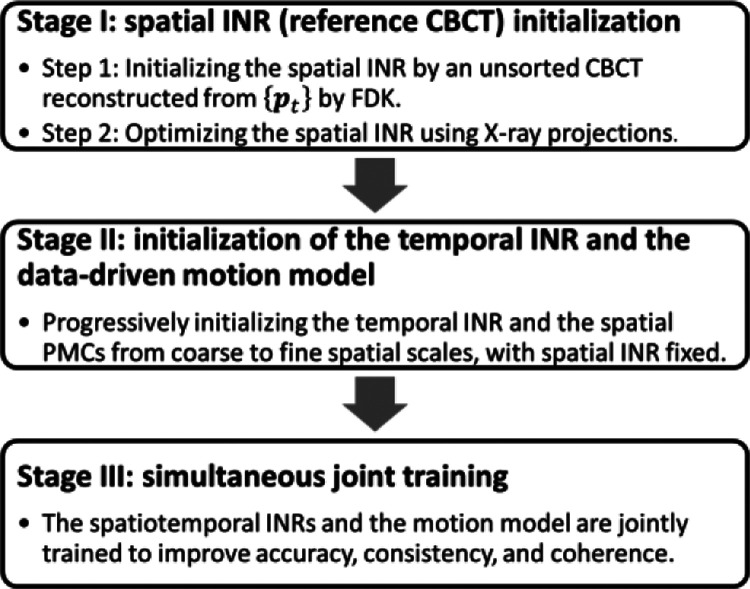
The three-staged progressive training strategy of PMF-STINR.

While different strategies have been introduced to mitigate the ill-posed spatiotemporal reconstruction problem in above sections, training the spatial and temporal INRs and the data-driven motion model simultaneously remains challenging, due to the spatiotemporal ambiguity and the limited sampling. To address this issue, a three-staged, progressive training strategy was developed for PMF-STINR (Fig. 4) on the foundation of the prior STINR framework [13]. The strategy progressively increases the learning complexity through three stages to help avoid the local optimum during the training. In Stage I, the spatial INR was initialized by a motion-averaged CBCT reconstructed from all available projections ${\left\{p_{t}\right\}}_{t=0}^{N_{p}-1}$. The approximate reference CBCT $I_{\text{approx}}(x)$ was reconstructed using the Feldkamp-Davis-Kress (FDK) algorithm [21]. The fidelity loss of this step was thus defined in the image domain:

(7) $$L_{\text{img }}=\frac{1}{N_{\text{voxel }}}\sum_{i=1}^{N_{\text{voxel }}}{\left\|INR_{\text{spa }}\left(x_{i}\right)-I_{\text{approx }}\left(x_{i}\right)\right\|}^{2},$$

where $N_{\text{voxel}}$ is the total number of voxels in the reference-frame CBCT, and $INR_{\text{spa}}$ denotes the spatial INR. As the full-projection reconstruction contains FDK-related artifacts, in the second step of Stage I, $INR_{spa}$ was instead optimized through a fidelity loss defined in the projection domain, similar to the conventional iterative forward-backward projection:

(8) $$L_{\text{prj}}^{a}=\frac{1}{N_{\text{pixel }}}\sum_{j=1}^{N_{\text{pixel }}}{\left\|𝒫\;INR_{\text{spa }}\left(x\right)-\left\{p_{t}\right\}\right\|}^{2},$$

where $N_{\text{pixel}}$ is the number of projection pixels. In addition to the fidelity loss, an image-domain L-1 regularization loss (total variation, TV) was also introduced in this step to promote the sparsity of the reference CBCT in its gradient domain:

(9) $$L_{TV}=\frac{1}{N_{\text{voxel }}}\sum_{i}\left|\nabla I_{\text{ref }}\left(x_{i}\right)\right|,$$

where ∇ denotes the gradient operator. The overall loss function for the second step of Stage I is then:

(10) $$L_{\text{tot}}^{I}=L_{\text{prj}}^{a}+\lambda_{TV}L_{TV}.$$

The value of $\lambda_{TV}$ was set to $1\times 10^{-3}$ via empirical searching, and the same value was used throughout the following Stages (II and III). The training epochs of the first and second steps of Stage I were 1,000 and 600, respectively.

In Stage II, the temporal INR and the data-driven motion model were initialized, with respect to the reference-frame CBCT ${INR}_{\text{spa}}$ obtained from Stage I. The spatial INR was frozen at Stage II to prevent the interplay between the spatial and temporal INRs. For the multiresolution motion model, the three PMCs of increasing resolutions were learned progressively. In detail, the motion model starts with learning only one PMC per direction, and the other two PMCs of increasing resolutions were progressively added into the learning process. When a finer-scale PMC was introduced into the learning, the coarse-scale PMCs were frozen without updating. The training on each scale used 100 epochs. The learning of Stage II used the projection-domain fidelity loss:

(11) $$L_{\text{prj}}^{b}=\frac{1}{N_{\text{pixel }}}\sum_{t}\sum_{j=1}^{N_{\text{pixel }}}{\left\|𝒫\;INR_{\text{spa }}\left(x+\sum_{i,k=1}^{3}INR_{\text{tem }}^{i,k}\left(t\right)\times e_{i,k}\left(x\right)\right)-p_{t}\right\|}^{2},$$

where $INR_{tem}^{i,k}(t)$ denotes the temporal INR output for the $i^{\text{th}}$ PMC along the $k^{\text{th}}$ direction, at queried time frame t. Besides the fidelity loss, a regularization loss was applied to the PMCs to remove the ambiguity in the partial separation shown in Eq. 3. The loss enforced the orthonormal condition on $\left\{e_{i}(x)\right\}$:

(12) $$L_{PMC}=\sum_{i=1}^{3}\left({\left\|e_{i}\right\|}^{2}-1+\sum_{j=i+1}^{3}e_{i}\cdot e_{j}\right),$$

where the inner product is defined in the Hilbert space of the PMCs. After initializing the finest-scale PMCs, the multiresolution PMCs were unfrozen and trained for an additional 50 epochs for fine-tuning before entering the next Stage (III). The overall loss function of Stage II was defined as

(13) $$L_{\text{tot}}^{II}=L_{\text{prj}}^{b}+\lambda_{TV}L_{TV}+\lambda_{PMC}L_{PMC}.$$

The value of $\lambda_{PMC}$ was set to 1 via empirical searching, and the same value was used throughout Stages II and III.

Stage III performs joint training in which all components in PMF-STINR were unfrozen, based on the same loss function as Stage II (Eq. 13). The joint training allows simultaneous image reconstruction and registration to improve the accuracy of the reference CBCT and the coherence of the solved intra-scan motion. The training in Stage III used a total of 2,000 epochs.

PMF-STINR was implemented via the PyTorch library and trained on a graphic processing unit (GPU) card (NVIDIA Tesla V100).We used the Adam optimizer for the three-staged training, and the learning rates were reset when the fidelity loss changed from the image domain to the projection domain in Stage I, and when entering the Stage II or III. The learning rates of the spatial INR were respectively $4\times 10^{-4}$ and $1\times 10^{-7}$ for the two steps of Stage I, and $1\times 10^{-8}$ and $5\times 10^{-9}$ for Stages II and III, respectively. The temporal INR and the B-spline motion model used the same learning rates, which were $2\times 10^{-3}$ and $2\times 10^{-4}$ for Stages II and III, respectively.

### C. Data Curation and Evaluation Schemes

A comprehensive evaluation was performed on PMF-STINR. It was first tested on the XCAT digital phantom via a simulation study [14]. Subsequently, it was tested on a dynamic thorax CIRS phantom (Computerized Imaging Reference Systems, Inc.) via a measurement study. Both XCAT and CIRS phantom studies provided ‘ground-truth’ images/motion for quantitative evaluations. Finally, PMF-STINR was tested on a multi-institutional dataset of real patient scans with various acquisition protocols/scanners to assess its robustness.

#### 1) The XCAT phantom study

**Fig. 5:**
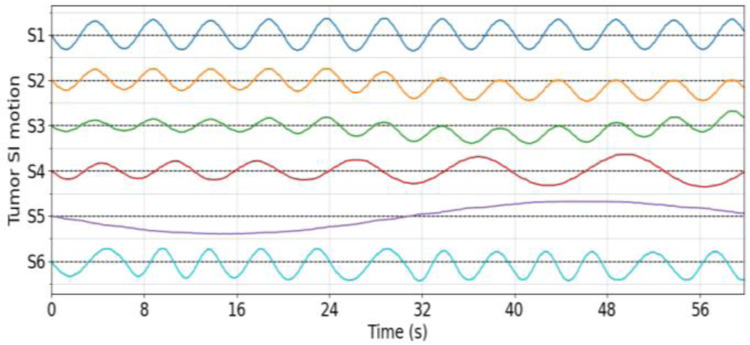
Lung tumor center-of-mass motion in the superior-inferior (SI) direction of the XCAT study. Horizontal dash lines indicate the abscissae of the six scenarios (S1–S6), and one ordinate tick equals 15 mm. See text for detailed description of the motion scenarios.

The simulated XCAT phantom covers the thoracic-abdominal portion of the anatomy, for a dimension of 128×128×64 voxels and an isotropic $4\times 4\times 4{\;mm}^{3}$ voxel size. A spherical lung tumor 30-mm in diameter was inserted into the lower lobe of the right lung for motion tracking and assessment. Six free-breathing scenarios (S1…S6) were simulated to assess the accuracy of PMF-STINR in reconstructing dynamic CBCTs to capture different motion variations (Fig. 5). S1 simulates the simplest scenario of a quasi-periodic breathing cycle (~5 s) with small amplitude variations. The average range of the tumor center-of-mass motion was about 13 mm. S2 includes a rapid baseline shift (~5 mm) in the middle of the scan (~30 s). S3 combines both breathing amplitude variations and baseline shifts. The breathing period of S4 is gradually increasing, along with the motion amplitude. S5 simulates a slow breathing or a fast-rotation scan where the acquisition contains only a single breathing cycle. This scenario is deemed extremely challenging for motion-sorting-based reconstruction algorithms, as the projection angles of the sorted phases will be limited to a small range. S6 combines variations of the breathing period, amplitude, and baseline shift.

Based on the dynamic XCAT volumes, cone-beam projections were simulated using the tomographic package (ASTRA toolbox [22]). The total scan time was set to 60 s, covering a 360° scan angle (6°/s gantry rotation speed). A total of $N_{t}=660$ projections were generated based on a frame rate of 11 fps to mimic a clinical 3D CBCT scan. Each projection contained 256×192 pixels for a pixel resolution of $1.6\times 1.6{\;mm}^{2}$.

The accuracy of the reconstructed dynamic CBCTs was quantitatively evaluated using the relative error (RE) and the structural similarity index (SSIM) [23]. RE was defined as

(14) $$RE=\frac{1}{N_{p}}\underset{t}{\sum }\sqrt{\frac{\sum_{i=1}^{N_{\text{voxel }}}{\left\|\overset{ˆ}{I}\left(x_{i},t\right)-I^{gt}\left(x_{i},t\right)\right\|}^{2}}{\sum_{i=1}^{N_{\text{voxel }}}{\left\|I^{gt}\left(x_{i},t\right)\right\|}^{2}}},$$

where $I^{gt}$ denotes the ‘ground-truth’ CBCTs. The accuracy of solved motion was evaluated by contour-based metrics: tumor center-of-mass error (COME) and Dice similarity coefficient (DSC). To calculate COMEs and DSCs, the lung tumors in the reference-frame CBCTs were contoured and propagated to other dynamic CBCTs by the motion fields solved by PMF-STINR, and compared with the ‘ground-truth’.

#### 2) The dynamic thorax phantom study

A dynamic thorax phantom (CIRS) was employed in a clinical measurement study to assess PMF-STINR. For CIRS, a spherical tumor with electron density similar to that of the phantom body was placed in the left lung. The tumor motion is driven by an actuator and can be customized. Six motion trajectories were used in the CIRS phantom study, including S1, S3, S4, and S5 from the XCAT study, and two additional irregular trajectories (S7 and S8). The peak-to-peak amplitudes in the superior-inferior (SI) direction of these trajectories ranged from 24 to 30 mm, and those in the anterior-posterior (AP) direction ranged from 0 to 10 mm.

For each of the motion scenarios, cone-beam projections of the dynamic phantom were acquired on a Varian VitalBeam system (Varian Medical Systems, Inc.) in the half-fan mode, with the phantom center aligned to the imaging isocenter. Each scan took about 1 min for a 360° scan angle, acquiring 894–896 projections. Due to the off-axis tumor location and the half-fan scan, the tumor was only visible in about half of the projections. The projections were acquired under a 125 kVp energy, with mAs of 15 mA/20 ms. Each projection had 1024×768 pixels with a $0.388\times 0.388{\;mm}^{2}$ pixel resolution. The projections were down-sampled to 256×172 before the reconstruction, and the size of reconstructed CBCTs was 200×200×68 with an isotropic resolution of $3\times 3\times 3{\;mm}^{3}$.

The solved tumor motion was compared against the programmed trajectories. Tumor COMEs in the AP, left-right (LR), and SI directions were individually evaluated. The Pearson correlation coefficient between the solved and the ‘ground-truth’ tumor motion trajectories was computed. Due to the half-fan scan geometry, the accuracy was evaluated only on the projection frames where the tumor was in the field-of-view.

#### 3) The patient study

PMF-STINR was further evaluated on a multi-institutional real patient dataset. Table I summarizes the imaging parameters of the patient study. The half-scan scans were reconstructed of a lower spatial resolution than the full-fan scans to accommodate the extended FOV. A total of 12 cone-beam projection sets from eight patients were curated from three sources. The MDACC data (P1…P3) were acquired by a Varian system in full-fan mode [3]. A slow-gantry acquisition scheme was used to cover a 200° scan angle. The scans took between 4.5–5.8 min, acquiring 1,653–2,729 projections. The SPARE data were taken from the SPARE Challenge [4] which evaluated 4D-CBCT reconstruction algorithms from sparse-view acquisitions in both full- and half-fan modes. The SPARE data contained 10 patients, and we selected four patients with clear anatomical structures that can be tracked in 2D projections for motion evaluation. The full- and half-fan scans were acquired from Elekta and Varian systems, respectively. For the SPARE data, each patient had two sets of projections: one was a fully-sampled scan, and the other was a down-sampled sparse-view set equivalent to a 1-min scan (patient ID ends with a suffix ‘S’). As in Table I, the sparse-view sets had much fewer projections. The UTSW data (P6) were acquired by a Varian system in half-fan mode. The scan time was about 1 min, covering a 360° scan angle.

Since the patient study had no ‘ground-truth’ 3D motion for evaluation, accuracy of the solved intra-scan motion was evaluated in re-projected 2D planes. Specifically, for each reconstructed dynamic CBCT, we re-projected it into a 2D digitally reconstructed radiograph (DRR) to match the corresponding cone-beam projection’s imaging geometry. Motion tracked on the cone-beam projections and the DRRs was then compared by two methods: **(1)**. Structure-based motion evaluation. The structures being tracked include diaphragm, tumor, and lung structure. For diaphragm tracking, the Amsterdam Shroud (AS) technique [24] was employed to highlight the motion-induced intensity variations on both the cone-beam projection sets and the re-projected DRR sets. From the view-consolidated Amsterdam Shroud image, motion of the diaphragm dome was extracted based on the sharp image contrast at the diaphragm boundary. The match between the diaphragm motion tracked on the original cone-beam projections and that tracked on the re-projected DRRs was evaluated by the Pearson correlation coefficient and the position difference. Since the diaphragm of P1 moved in-and-out of the field-of-view during the scan, instead of the diaphragm, a high-density lung nodule was tracked with a similar approach as diaphragm tracking. In addition, diaphragm of P3 was barely visible in the projections, so the respiratory motion trajectory was extracted from the AS images using a high-contrast lung feature. **(2).** Feature point-based motion evaluation. The second method automatically tracked corresponding feature points from both cone-beam projections and DRRs for motion comparison, without using the AS images [9, 25]. Specifically, it first automatically and independently extracted image feature points from cone-beam projections based on local intensity variations, with the selected points typically at the boundaries of anatomic features. Next, corresponding feature points in these projections were identified and selected, and their motion trajectories across multiple frames were tracked, using the M-estimator sample consensus algorithm [26]. Finally, the corresponding feature points in the DRRs were localized and tracked by a correlation coefficient-based searching algorithm for comparison. The feature point motion difference (localization error, LE) between the cone-beam projections and DRRs was evaluated by [9]:

(15) $$LE=\sqrt{\frac{1}{N_{p}}\sum_{p}\frac{1}{M_{p}}\sum_{q}{\left(z_{pq}^{cb}-z_{p}^{DRR}\right)}^{2}},$$

where $M_{p}$ denotes the number of feature points in the $p^{\text{th}}$ cone-beam projection, $z_{pq}^{cb}$ denotes the tracked location of the $q^{\text{th}}$ feature point in the $p^{\text{th}}$ cone-beam projection, and $z_{p}^{DRR}$ is the tracked location of the corresponding point in the $p^{\text{th}}$ DRR. Due to the limitations of tracking in 2D planes with rotating projection angle, we only calculated LE along the SI direction, which is the dominant direction of respiratory motion.

#### D. The comparison study

To evaluate the benefit of the prior model-free approach employed by PMF-STINR, it was compared with the previously-published STINR framework [13] in which the online-learned, data-driven motion model was replaced by a pre-defined, PCA-based motion model [9, 27–29]. Like the data-driven motion model, the PCA-based motion model yields a basis set of principal motion components to explain the motion fields (c.f., Eq. 3). However, the PCA-based motion model is not learned/optimized but generated prior to the reconstruction as a prior motion model. We performed this comparison study on the XCAT dataset with known ‘ground-truth’. Specifically, prior to the STINR reconstruction, we derived a PCA-based motion model from the XCAT cone-beam projections for each motion scenario (S1…S6, Fig. 5). The cone-beam projections of each motion scenario were first sorted and binned into 10 respiratory phases, except for scenario S5. For S5, we sorted the projections into 5 phases, as the single-cycle scenario yielded substantial limited-angle artifacts if 10 phases were used, which led to highly inaccurate PCA motion modeling. We reconstructed 4D-CBCTs from the phase-sorted projections using the FDK algorithm. Deformable image registrations between the end-exhale phase and the other phases were performed to obtain the inter-phase DVFs for each motion scenario, using Elastix [30]. Finally, we derived principal motion components from the inter-phase DVFs using PCA for each motion scenario, and fed them as a known motion model into the STINR reconstruction. This PCA-based variant was referred as PCA-STINR.

We compared the accuracy of the reconstructed dynamic CBCTs and the solved intra-scan motion between PCA-STINR and PMF-STINR. A Wilcoxon signed-rank test was performed to evaluate the statistical significance between the two methods.

## III. Results

### A. The XCAT Phantom Study

**Fig. 6:**
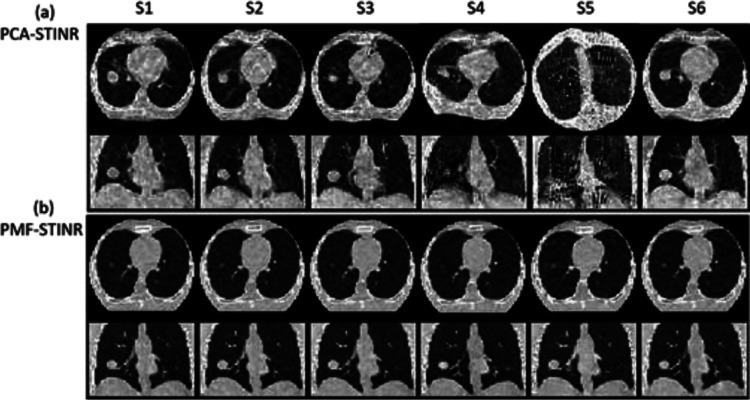
Comparison of reference-frame CBCTs reconstructed by (a) PCA-STINR and (b) PMF-STINR for the six motion scenarios (S1–S6) in the axial and coronal views.

Figure 6 compares the reconstructed reference-frame CBCTs of PCA-STINR and PMF-STINR for the XCAT study. PMF-STINR presented consistently better image quality in terms of the contrast, shape, and intensity distribution of the anatomy, across the six motion scenarios. PMF-STINR shows robustness to different motion variations and irregularities. Compared with PMF-STINR, PCA-STINR suffers from the inaccuracy of the pre-defined PCA-based motion model, which was extracted from artifacts-ridden phase-sorted 4D-CBCT reconstructions (i.e., sparse-sampling artifacts, blurriness from the intra-phase residual motion, and limited-angle artifacts as S5). Unlike PMF-STINR, where the principal motion components were learned and optimized on the fly simultaneously with the CBCT reconstruction, the PCA-STINR had to rely on the inaccurate PCA motion model throughout the image reconstruction, which negatively impacted the reference-frame CBCT reconstruction and intra-scan DVF optimization. Table II presents the quantitative metrics showing the accuracy of reconstructed dynamic CBCTs, using the ‘ground-truth’ XCAT volumes for reference. All Wilcoxon signed-rank tests had p-values < 10^−3^.

**Fig. 7:**
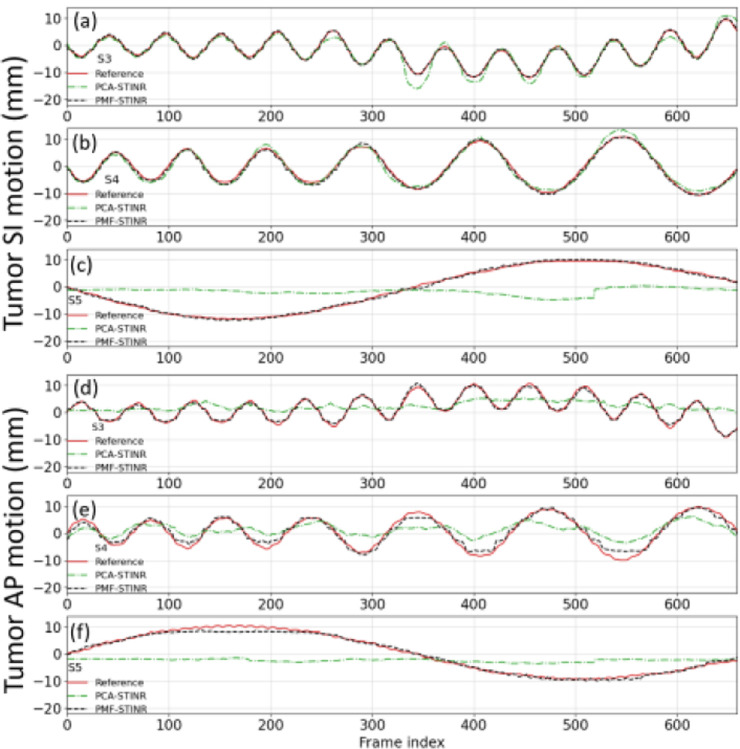
Comparison of tumor SI (a–c) and AP (d–f) trajectories of S3–S5, between PMF-STINR, PCA-STINR, and the ‘ground-truth’ (reference).

Figure 7 compares the lung tumor center-of-mass motion (SI and AP directions) solved by PCA-STINR and PMF-STINR for the XCAT study. Due to space limitations, only scenarios S3–S5 featuring various motion irregularities are presented; similar trends can be observed from the remaining scenarios. Table III shows the lung tumor COME (calculated in 3D) and DSC of the six motion scenarios. PMF-STINR showed consistently better results, achieving sub-millimeter accuracy on average.

### B. The Dynamic Thorax Phantom Study

**Fig. 8:**
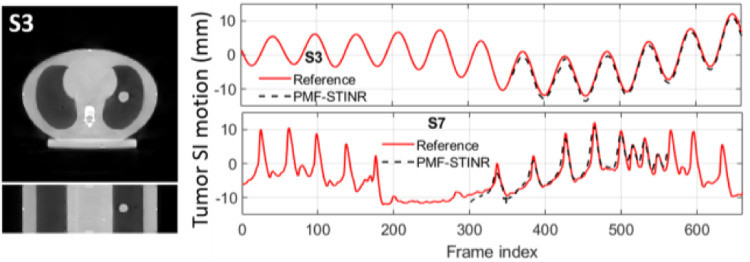
(Left) An example reference-frame CBCT reconstructed by PMF-STINR for the CIRS study (S3). (Right) Comparison between the programmed motion curves (reference) and the solved curves by PMF-STINR, for scenarios S3 and S7. For PMF-STINR, the tumor trajectory was only tracked for frames where the tumor was in the field-of-view.

Figure 8 presents the reconstructed reference-frame CBCT of PMF-STINR for scenario S3, and the comparison between the programmed motion curves and the solved curves (SI direction) by PMF-STINR for the CIRS study (S3 and S7). The PMF-STINR accurately captured the intra-scan motion of the tumor for both regular and irregular motion scenarios. Table IV presents the tumor center-of-mass localization errors in the LR, AP, and SI directions and the Pearson correlation coefficients between the solved and the ‘ground-truth’ SI trajectories.

### C. The Patient Study

**Fig. 9:**
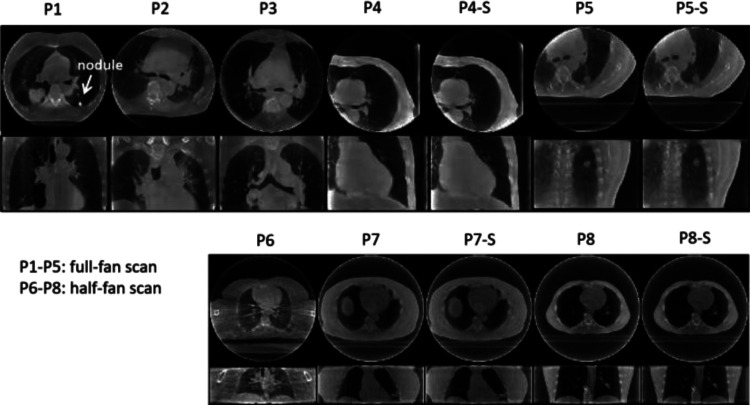
Reference-frame CBCTs reconstructed by PMF-STINR for the patient study. The upper and lower rows show the reconstructions from full- and half-fan scans, respectively.

**Fig. 10:**
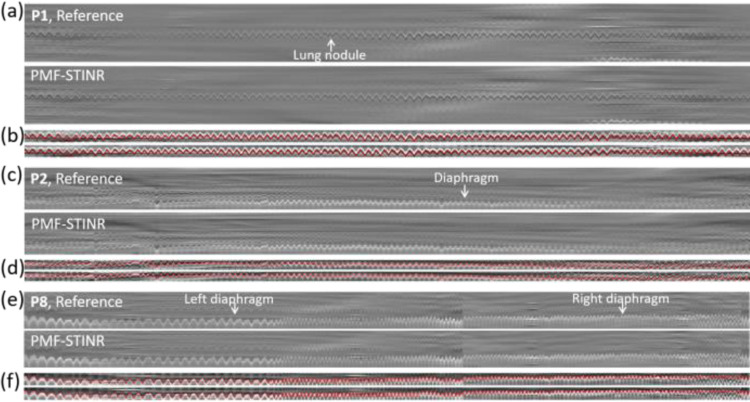
Comparison between the reference and the solved motion trajectories, respectively extracted from cone-beam projections and the DRRs via the Amsterdam Shroud (AS) method. (a–d) P1 and P2 were scanned by the full-fan mode where the tracking objects (P1: lung nodule, P2: diaphragm) were visible in all projections. (e–f) P8 was scanned by the half-fan mode where the tracked objects (diaphragms) were only visible in parts of the projections. Thus, the left and right diaphragms of P8 were tracked consecutively based on their visibility. Figs. 10-a&c&e show the AS images derived from the projections and DRRs. Figs. 10-b&d&f show the vertically cropped, z-score normalized regions containing the tracked objects, on which the motion trajectories (red lines) were extracted for quantitative evaluation.

Figure 9 presents the reference-frame CBCTs reconstructed for the patient study. The reference-frame CBCTs of the fully- and sparsely-sampled acquisitions (P4, P5, P7, and P8) are comparable in image quality, showing PMF-STINR allows dynamic CBCT reconstruction even from sparsely-sampled 3D CBCT scans (~300 cone-beam projections in total, Table I). The high-density lung nodule of P1 used for motion tracking and evaluation were highlighted (Sec.II.C.3). Figure 10 compares the extracted motion trajectories by the AS method (10-a&b: lung nodule, 10-c&d&e&f: diaphragm). The red curves overlaid on 10-b&d&f indicate the extracted motion curves based on the AS image.

Table V shows the accuracy of the solved motion, using both the AS and the feature point tracking methods. Overall, PMF-STINR achieved accurate structure and feature point localization in the dynamic sequence, as evaluated by the counterpart reference signals extracted from the corresponding cone-beam projections. The localization errors presented here were calculated in projected 2D planes, and included the magnification factor (~1.5) due to the imaging trajectory. On average a sub-millimeter accuracy was achieved by PMF-STINR-solved motion, after accounting for the factor.

## IV. Discussion

In this study, we proposed PMF-STINR (Fig. 1), an INR-based framework to address the dynamic CBCT reconstruction challenge. Without relying on any prior anatomical or motion model, PMF-STINR reconstructs dynamic CBCTs and solves the intra-scan motion simultaneously from the cone-beam projections via a ‘one-shot’ learning scheme. PMF-STINR decouples the ill-posed spatiotemporal inverse problem of dynamic CBCT reconstruction into learning a spatial INR module (Fig. 2), a temporal INR module (Fig. 3), and a trainable B-spline-based, data-driven motion model, and develops a progressive learning strategy (Fig. 4) that is able to reconstruct dynamic CBCTs from conventional 3D CBCT scans with only ~300 projections (Table 1). Compared with the prior STINR framework, PMF-STINR achieves substantially higher reconstruction and motion tracking accuracy by the online-learned motion model, and does not require any prior motion sorting and binning (Figs. 6 and 7, Tables II & III). The use of the hash encoding, compared to the Fourier frequency encoding, enables faster computation and allows lightweight MLPs to be used for the INRs. The comprehensive evaluation of PMF-STINR on the XCAT (Figs. 6 and 7, Tables II & III), the CIRS (Fig. 8, Table IV), and the patient datasets (Figs. 9 and 10, Table V) demonstrated its robustness to varying anatomical, motion, and imaging variations, a substantial advantage over previous deep learning-based models which require extensive training and are susceptible to out-of-distribution shifts. The robustness and adaptability of PMF-STINR indicate a high clinical translation potential.

Whereas the spatial and temporal INRs were lightweight and learning efficient, currently PMF-STINR took about 4 hours to reconstruct a sequence of 2000-frame dynamic CBCTs. A major speed bottleneck was the cone-beam projector from the ASTRA toolbox [22], which was optimized for projecting multiple DRRs from the same CBCT in parallel. However, PMF-STINR only requires a single DRR at a gantry angle for a dynamic CBCT, and thus the DRRs of different dynamic CBCTs were sequentially projected in the current framework. To reduce the reconstruction time, the high throughput of a GPU can be leveraged by parallelizing a cone-beam projector to generate DRRs of different CBCTs simultaneously.

In the present study, PMF-STINR was assessed for solving the respiratory motion of the thoracic-abdominal region. The proposed framework, in theory, can handle other types of anatomical motions, such as the cardiac beats and peristalsis. However, for motion fields involving multiple modes of anatomical motions with disparate temporal and spatial scales (e.g., respiratory and cardiac motions have distinct periods of about 3–5 s and 0.8–1 s, respectively), the PMF-STINR framework may warrant additional modifications to effectively describe the multi-mode motion field. It is possible to describe a multi-mode and multi-scale DVF by increasing the number of PMCs along each direction, or further introducing region-focused PMCs. The current motion model used a uniform B-spline grid to cover the entire reconstructed volume. For improvement, a non-uniform grid can be used wherein the grid density will automatically adapt to the involved anatomical locations. Further investigations and developments of PMF-STINR are warranted to evaluate and potentially improve its versatility and adaptability for different motion types and scenarios.

Currently, PMF-STINR was designed for the retrospective reconstruction of dynamic CBCTs, preventing it from solving time-resolved motion in real-time. However, with additional modifications to the framework, PMF-STINR may leverage the obtained knowledge of the reference-frame CBCT and the learned motion model to achieve real-time imaging and tumor localization. Currently, the temporal INR takes a frame index as input and outputs the temporal coefficients of the PMCs at the queried time point. To adapt the framework for real-time imaging, the input can be replaced by motion-related image features extracted from a cone-beam projection by a DL network. This modification may allow the temporal INR to learn the mapping from the extracted imaging features to the coefficients of PMCs. After the network training, the temporal INR may directly use real-time-acquired imaging features to infer the PMC coefficients and compose real-time DVFs to represent instantaneous motion. Such modifications towards real-time imaging are currently under investigation with future reports anticipated.

## Figures and Tables

**Table I:** Summary of imaging parameters of the patient study.

Patient ID^a^	Source^b^	Vender	Scan mode	Projection size^c^	Pixel size (mm^2^)	kVp/mA/mS	SAD^d^ (mm)/SDD (mm)	Reconstructed CBCT voxels	Voxel size (mm^3^)
P1	MDACC	Varian	Full fan	512×384×1983	0.776×0.776	120/80/25	1000/1500	200×200×100	2×2×2
P2	MDACC	Varian	Full fan	512×384×2729	0.776×0.776	120/80/25	1000/1500	200×200×100	2×2×2
P3	MDACC	Varian	Full fan	512×384×1653	0.776×0.776	120/80/25	1000/1500	200×200×100	2×2×2
P4	SPARE	Elekta	Full fan	512×512×1015	0.8×0.8	125/20/20	1000/1536	200×200×100	2×2×2
P4-S	SPARE	Elekta	Full fan	512×512×340	0.8×0.8	125/20/20	1000/1536	200×200×100	2×2×2
P5	SPARE	Elekta	Full fan	512×512×1005	0.8×0.8	125/20/20	1000/1536	200×200×100	2×2×2
P5-S	SPARE	Elekta	Full fan	512×512×340	0.8×0.8	125/20/20	1000/1536	200×200×100	2×2×2
P6	UTSW	Varian	Half fan	1024×768×895	0.388×0.388	125/15/20	1000/1500	206×206×68	3×3×3
P7	SPARE	Varian	Half fan	1006×750×2416	0.388×0.388	120/20/20	1000/1500	200×200×68	3×3×3
P7-S	SPARE	Varian	Half fan	1006×750×679	0.388×0.388	120/20/20	1000/1500	200×200×68	3×3×3
P8	SPARE	Varian	Half fan	1006×750×2918	0.388×0.388	120/20/20	1000/1500	200×200×68	3×3×3
P8-S	SPARE	Varian	Half fan	1006×750×677	0.388×0.388	120/20/20	1000/1500	200×200×68	3×3×3

a Patient ID with a suffix ‘S’ indicates a sparsely-sampled set extracted from the fully-sampled one.

b MDACC: MD Anderson Cancer Center [3]. SPARE: SPARE Challenge [4]. UTSW: University of Texas Southwestern Medical Center.

c $width\;\left(in\;pixel\;number\right)\times \;height\;\left(in\;pixel\;number\right)\times N_{p}\;(number\;of\;projections)$.

d SAD: source-to-axis distance. SDD: source-to-detector distance.

**Table II:** Accuracy of the reconstructed dynamic CBCTs of the XCAT study, in terms of the relative error and the structural similarity index (SSIM). The results are presented as mean ± S.D.. The arrows are pointing to the direction of improved accuracy.

Motion scenario	Relative error ↓		SSIM ↑	
	PCA-STINR	PMF-STINR	PCA-STINR	PMF-STINR
S1	0.244±0.021	**0.149±0.008**	0.890±0.006	**0.984±0.002**
S2	0.264±0.034	**0.143±0.016**	0.905±0.010	**0.985±0.004**
S3	0.254±0.040	**0.137±0.016**	0.912±0.012	**0.986±0.004**
S4	0.279±0.029	**0.168±0.020**	0.865±0.007	**0.979±0.006**
S5	0.365±0.034	**0.170±0.010**	0.790±0.011	**0.978±0.003**
S6	0.251±0.021	**0.149±0.010**	0.892±0.006	**0.984±0.003**

**Table III:** Lung tumor localization accuracy of the XCAT study. The results are presented in terms of mean ± S.D. The arrows are pointing to the direction of improved accuracy.

Motion scenario	COME (mm) ↓		DSC ↑	
	PCA-STINR	PMF-STINR	PCA-STINR	PMF-STINR
S1	2.2±1.5	**0.8±0.4**	0.86±0.05	**0.91±0.02**
S2	4.2±2.6	**0.8±0.4**	0.80±0.09	**0.92±0.03**
S3	3.5±2.4	**0.8±0.3**	0.82±0.10	**0.93±0.03**
S4	3.9±1.8	**1.3±0.6**	0.78±0.07	**0.91±0.03**
S5	10.1±4.4	**1.0±0.5**	0.43±0.12	**0.90±0.02**
S6	3.4±1.8	**0.9±0.4**	0.82±0.05	**0.90±0.02**

**Table IV:** Lung tumor localization accuracy by PMF-STINR for the dynamic thorax phantom study. The results are presented in terms of mean ± S.D.. The arrows are pointing to the direction of improved accuracy.

Motion scenario	Pearson correlation coefficient ↑	Tumor localization error ↓		
		LR (mm)	AP (mm)	SI (mm)
S1	0.997	0.4±0.4	1.1±0.7	1.2±0.7
S3	0.999	0.4±0.2	1.0±0.7	1.0±0.3
S4	0.999	0.5±0.6	1.0±0.6	1.0±0.4
S5	0.999	0.3±0.3	1.1±0.9	1.0±0.2
S7	0.959	0.4±0.4	0.2±0.2	1.1±1.1
S8	0.979	0.2±0.3	1.1±0.4	0.9±0.6

**Table V:** Accuracy of the solved intra-scan motion by PMF-STINR for the patient study. The results are presented in terms of mean ± S.D.. The arrows are pointing to the direction of improved accuracy.

Patient ID	Pearson correlation coefficient ↑	localization error (mm) ↓	
		AS	Feature point tracking
P1	0.968	1.1±1.1	0.5±0.7
P2	0.987	1.1±1.2	1.1±1.1
P3	0.943	1.1±1.3	0.9±0.9
P4	0.976	1.1±1.0	1.7±1.1
P4-S	0.955	1.3±1.5	1.6±1.3
P5	0.978	2.7±2.2	1.2±1.3
P5-S	0.978	2.3±2.1	1.1±1.6
P6	0.987	1.4±1.2	0.7±0.8
P7	0.992	1.7±1.4	1.9±1.6
P7-S	0.992	1.7±1.5	1.8±1.6
P8	0.992	1.4±1.1	1.9±1.4
P8-S	0.990	1.8±1.6	2.1±1.8
